# Imperfect but Hopeful: New Advances in Soil Pollution and Remediation

**DOI:** 10.3390/ijerph191610164

**Published:** 2022-08-16

**Authors:** Liping Li, Lanfang Han, Aiju Liu, Fayuan Wang

**Affiliations:** 1 School of the Environment, Henan University of Technology, Zhengzhou 450001, China; 2 School of Ecology, Environment and Resources, Guangdong University of Technology, Guangzhou 510006, China; 3 School of Resources and Environmental Engineering, Shandong University of Technology, Zibo 255049, China; 4 College of Environment and Safety Engineering, Qingdao University of Science and Technology, Qingdao 266042, China

Soil is the most important resource for plant growth and human survival, supporting agricultural production and human habitation. However, due to unreasonable anthropogenic activities, soil receives a large amount of hazardous and toxic materials, including common contaminants such as toxic metal(loid)s and organic contaminants, and emerging contaminants with uncertain hazards and toxicity, posing potential risks for the soil ecosystems, food security, and human health. Thus, it is of great importance to recognize the environmental behaviors and fate of contaminants, and to improve remediation techniques. However, the environmental behaviors of contaminants have high complexity and spatial heterogeneity in soil environments. It is really challenging to illustrate soil pollution processes and to successfully remediate polluted soil. In particular, highly efficient, low-cost, and environmentally friendly soil remediation techniques need to be developed urgently.

In this Special Issue (SI), new advances in soil pollution and remediation were reported. Ten papers were published, six ones reporting the occurrence, environmental behaviors, and risks of contaminants, and the other four focusing on the remediation techniques of polluted soils. A series of contaminants were targeted, including toxic metal(loid)s (e.g., Pb, As, Sb, and multi-metals), organic contaminants (e.g., organochlorine pesticides, phenanthrene (PHe), and petroleum), and antibiotics (e.g., sulfadiazine). Both single and combined pollution scenarios were involved. Below, we summarized the main findings of these studies.

Non-ferrous metal mining and smelting is one of the main sources of heavy metal pollution in the environment. Because of the co-existence of trace elements in the mining ore, non-ferrous metal mining and smelting often results in the accumulation of multiple heavy metals in the soil. It is necessary to identify the sources of different metals. For sources with different Pb isotopic fingerprints, the Pb isotopic ratios of the sample and end member materials (such as ore and unpolluted soil) can be used for source apportionment of the Pb in the soil samples. Tang et al. [1] investigated the distribution of Cu, Pb, Zn, Cr, Ni, Cd, As, and Hg in soils and sediments near former gold mines in Beijing and Pb source apportionment with sample Pb isotopic information. They found that Cd and Pb had the greatest pollution indexes (as contamination factor in this paper) among all the metals. The results highlight the need for reasonable management of such deserted sites with high heavy metal accumulation. Cai and Li [2] studied the potential ecological risk, source, and input flux of heavy metals in the Hebei plain of northern China, and detected the accumulation of As, Cu, Cd, and Zn. With the input flux calculation, atmospheric dry and wet deposition were interestingly found to contribute more to soil pollution than the usage of fertilizer or irrigation water. Their results provide valuable data for controlling and remedying heavy metals polluted with agricultural soils.

Differently, Chen et al. [3] investigated the occurrences of organochlorine pesticides (OCPs) in karst soil by analyzing 25 OCPs in the karst soils near the Three Gorges Dam, China. In this karst area, p,p’-DDT and mirex were the most abundant OCPs, with different concentrations in spatial distribution as the land use type and the water transport. These OCPs were mainly derived from current agrochemical use and current veterinary use in the study area. OCPs are among typical persistent organic pollutants (POPs) with high toxicity. More attention should be paid to the issues of their illegal uses and bioaccumulation via the food chain.

Arsenic is one of the most toxic and widespread metalloids in the natural environment, which was targeted by two studies in this SI. Yin et al. [4] analyzed the effects of microorganisms, low-molecular-weight organic acid salts, and phosphates on the migration of As in unrestored and nano zero-valent iron (nZVI)-restored soil. They showed that microorganisms suppressed As release in both unrestored and restored soil. Meng et al. [5] explored As stress-induced response of three leafy vegetables, i.e., garland chrysanthemum (*Chrysanthemum coronarium* L.), spinach (*Spinacia oleracea* L.), and lettuce (*Lactuca sativa* L.), and found that the high tolerance of garland chrysanthemum was mainly ascribed to the low transport of As from the roots to the shoots, the high activity of antioxidant enzymes (*superoxide dismutase*, *glutathione peroxidase*, and *catalase*), and the abundant phytochelatins in the roots.

Antibiotics are identified as an emerging contaminant. The co-existence of antibiotics with heavy metals in the soil environment is attracting increasing attention. Xu et al. [6] investigated the adsorption behavior of sulfadiazine (SDZ) in paddy soils, and found that the changes in soil pH and ion concentration decreased the adsorption of SDZ on soil components, while dissolved organic carbon (DOC) facilitated the adsorption of SDZ in paddy soils, but the effect of co-existent Cu^2+^ was greatly dependent on the type of soil components. Their results confirmed that complexation may not be the only form of Cu^2+^ and SDZ co-adsorption in paddy soils.

This SI also brings some exciting advances in soil remediation. Biochar is a promising amendment used for the remediation of soils polluted with heavy metals and organic contaminants. Li et al. [7] reported the simultaneous stabilization/solidification of heavy metals and PHe by β-cyclodextrin-modified biochar. This functional material has abundant oxygen-containing functional groups on the surface and a porous structure with a large specific surface area, thus greatly increasing the retention of Cd, Cr, Cu, Pb, and PHe in soil. Lin et al. [8] reported an environmentally friendly soil remediation method with biochar/graphite carbon nitride (BC/g-C_3_N_4_), and indicated its technical feasibility of remediating petroleum-contaminated soil. BC/g-C_3_N_4_ facilitated the degradation by reducing recombination and better electron–hole pair separation. After treatment with BC/g-C_3_N_4_, the removal rates of nC_13_-nC_35_ were above 90% in the contaminated soil. In sum, the BC/g-C_3_N_4_ composites can effectively remedy organic contaminated soil.

In recent years, Sb pollution in the soil environment is drawing more attention. For the immobilization of Sb in polluted soils, a dilemma is that Sb existed as anions, while co-existing Cd and Pb ions are positively charged; thus, when a positively charged amendment can immobilize Sb in soil, it can also increase the availability and mobility of cationic contaminants in the soil. Thus, methods which can reduce the availability of both cations and anions are needed for Sb-contaminated soils. For this purpose, a combination of different additives may be an ideal choice. Wang et al. [9] studied the immobilization of Sb, Cu, and Zn with FeSO_4_ + Al(OH)_3_, and found that 5% FeSO_4_ + 4% Al(OH)_3_ was effective for the stabilization of Sb and co-occurring metals. However, for practical purposes, these dosages are still too high. In addition to the high cost, a high dosage of amendments may alter soil properties (such as texture, soil permeability, and phyto-availability of microelements), causing unexpected ecological risks for soil biota and plants.

A comprehensive evaluation of soil remediation technologies is of critical importance to the optimization and selection of proper technology for contaminated sites. Thermal desorption is an effective method to remediate sites contaminated with organic contaminants. Li et al. [10] developed a methodology to comprehensively evaluate three ex situ thermal desorption processes. The evaluation indicators included 20 qualitative and quantitative indicators covering technical, environmental, resource, economic, and social aspects. Their study provides a novel evaluation approach for the application of soil remediation technology. However, the applicability of this method needs to be verified in more case studies.

Due to their complexity of the soil environment, many contaminants are difficult to remove or degrade readily. Current techniques based on physical, chemical, and biological principles have some disadvantages, e.g., high price, environment unfriendliness, secondary pollution, and long processing time. While optimizing traditional techniques, new remediation materials and techniques should be developed. In addition, diverse emerging contaminants have been observed in the soil environment, including agricultural ecosystems [11,12]. However, their occurrence, environmental behaviors, fate, ecotoxicology, and potential risks are yet to be fully recognized, not to mention the remediation technology for their removal. Thus, we call for more efforts on emerging contaminants in the soil, particularly developing effective remediation techniques.
